# Artificial intelligence application for rapid fabrication of size-tunable PLGA microparticles in microfluidics

**DOI:** 10.1038/s41598-020-76477-5

**Published:** 2020-11-11

**Authors:** Safa A. Damiati, Damiano Rossi, Haakan N. Joensson, Samar Damiati

**Affiliations:** 1 Department of Pharmaceutics, Faculty of Pharmacy, King Abdulaziz University (KAU), Jeddah, 21589 Saudi Arabia; 2 Blacktrace Holdings Ltd (Dolomite Microfluidics), Royston, SG8 5TW UK; 3 Division of Protein Science, KTH Royal Institute of Technology, Stockholm, Sweden; 4 Novo Nordisk Foundation Center for Biosustainability at KTH, Stockholm, Sweden; 5 Department of Biochemistry, Faculty of Science, King Abdulaziz University (KAU), Jeddah, Saudi Arabia; 6 Division of Nanobiotechnology, Department of Protein Science, Science for Life Laboratory, School of Engineering Sciences in Chemistry, Biotechnology and Health, KTH Royal Institute of Technology, Stockholm, Sweden

**Keywords:** Biotechnology, Biomaterials

## Abstract

In this study, synthetic polymeric particles were effectively fabricated by combining modern technologies of artificial intelligence (AI) and microfluidics. Because size uniformity is a key factor that significantly influences the stability of polymeric particles, therefore, this work aimed to establish a new AI application using machine learning technology for prediction of the size of poly(d,l-lactide-co-glycolide) (PLGA) microparticles produced by diverse microfluidic systems either in the form of single or multiple particles. Experimentally, the most effective factors for tuning droplet/particle sizes are PLGA concentrations and the flow rates of dispersed and aqueous phases in microfluidics. These factors were utilized to develop five different and simple in structure artificial neural network (ANN) models that are capable of predicting PLGA particle sizes produced by different microfluidic systems either individually or jointly merged. The systematic development of ANN models allowed ultimate construction of a single in silico model which consists of data for three different microfluidic systems. This ANN model eventually allowed rapid prediction of particle sizes produced using various microfluidic systems. This AI application offers a new platform for further rapid and economical exploration of polymer particles production in defined sizes for various applications including biomimetic studies, biomedicine, and pharmaceutics.

## Introduction

Artificial intelligence (AI) has gained substantial recognition in numerous fields, such as pharmaceutics^1,2^, engineering^3^, education^4^, and many others^5–7^. Machine learning (ML) is a type of AI in which computers learn and ultimately perform tasks. One machine learning approach is artificial neural networks (ANNs) which are inspired by biological neurons. The typical ANN structure consists of three layers: input, hidden, and output layer. The process of ANN learning occurs through iterative representation of examples. The inputs are multiplied by connection weights. The information is then summed and transferred to the hidden layer using certain activation functions which ultimately send the results to the output layer^8^. ANNs are powerful machine learning tools for modelling nonlinear relationships which are frequently encountered in diverse research areas and industrial settings^9,10^.

AI can be utilized in synthetic bioarchitecture to design biosynthetic models that mimic natural biological membranes. Biomimicry facilitates the systematic investigations of biological membranes complexities and heterogeneities and supports generation of potential platforms for drug delivery^11^. A number of biomimetic membrane models have been developed over the last century such as Langmuir monolayers, supported lipid membranes, and vesicles^12^. Polymeric vesicles are promising artificial cell models with several advantages such as hydrophilicity, high stability, controllable physicochemical properties, prolonged circulation time, controlled release properties, and low cost^13–15^. Poly(d,l-lactide-co-glycolide) (PLGA) is a widely used synthetic polymer that is approved by the FDA due to its biosafety, biocompatibility, and biodegradability^16^. Currently, there are about 20 PLA/PLGA-based products approved by FDA and the European Medicines Agency (EMA) that are available on market and have been used as delivery vehicles for drugs, proteins and numerous macromolecules^17–19^.

Generation of uniform particles with well-controlled properties is a challenging task. Microfluidic techniques address limitations of bulk methods. Microfluidic devices enable the production of microparticles with high monodispersity, precisely tunable structures, and superior encapsulation efficiency^20–26^. Although the microfluidic generation method supports on-site and on-demand particle production, these methods usually involve extensive laboratory optimization. Further, it is difficult to predict the size of PLGA droplets and the particles that result from their drying based on non-dimensional parameters because such predictions require prior determination of fluid properties such as viscosity and surface tension which are difficult to ascertain^27^. Therefore, such predictions can be much more conveniently made in silico using AI technology. A number of studies have developed ML models to predict droplet microfluidics parameters including nanoprecipitation of drug particles^28^, droplet stability^29^, as well as fluid and flow parameters^30^.

**Figure 1:**
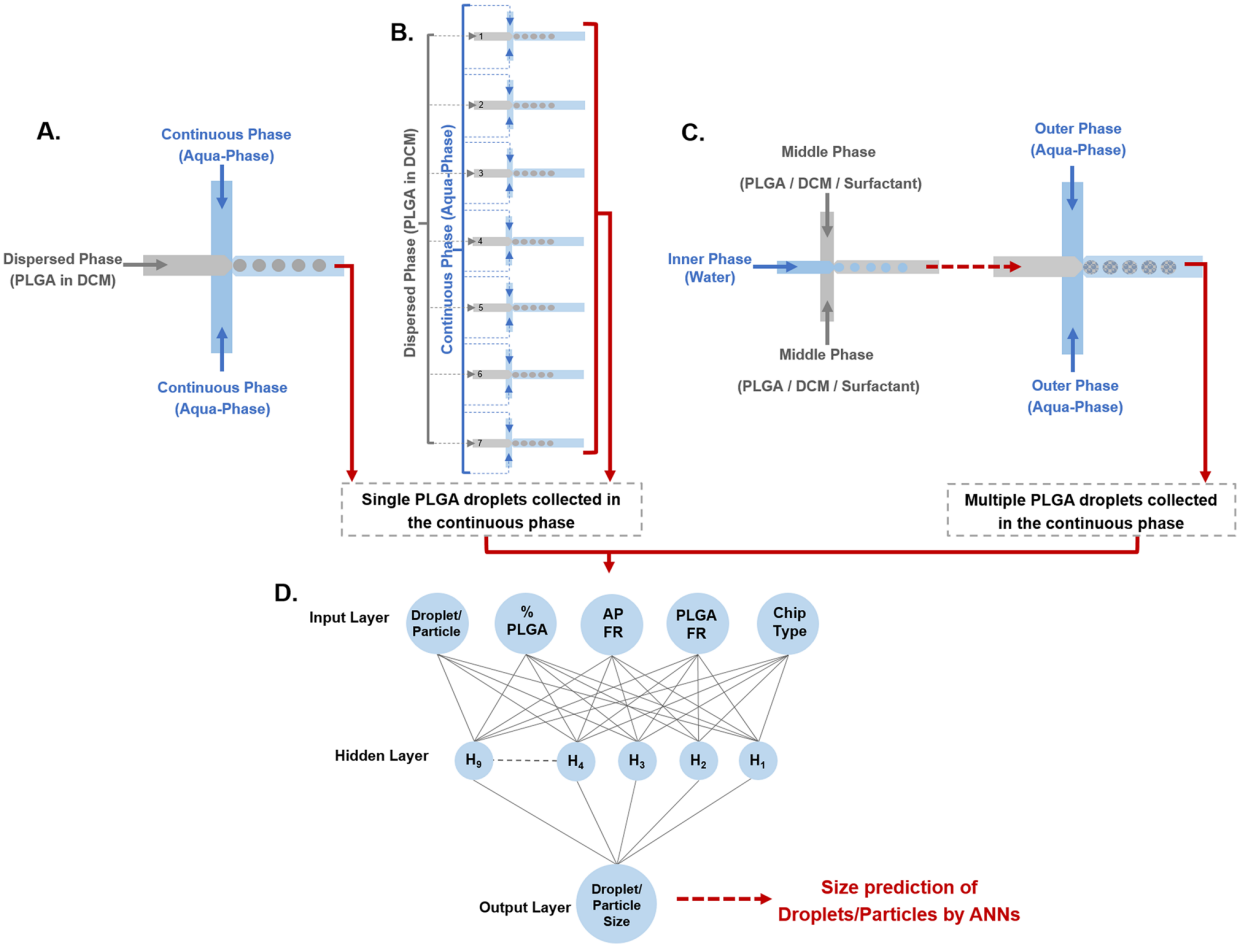
Schematic illustration of the generation of monodisperse PLGA droplets either in a single emulsion format by single junction devices (**A**) or microfluidic devices with seven parallel junctions (**B**), or in a multiple emulsion format (**C**) by two sequentially coupled devices. Generated droplets were imaged at the orifice of the flow-focusing region in the microfluidic chips. Data generated were thereafter used to train ANN models. Schematic of one of the developed models (ANN-ABC) (**D**). *AP* aqueous phase, *FR* flow rate.

In the current study we aimed to develop an in silico model using ANNs to predict both the size of PLGA droplets and the particles resulting from their drying, which are key to microfluidics production of polymeric microparticles. Whereas previous studies were limited to the prediction of parameters of a single microfluidic system, in this study we developed ANN predictive models for droplet and particle sizes resulting from three diverse microfluidic systems. We developed individual ANN models for each device as well as jointly merged ANN models encompassing two and three microfluidic devices. The microfluidic devices generated highly monodisperse particles with defined sizes either in single or multiple emulsion formats (Fig. 1). Subsequently, the experimental data together with a set of parameters including polymer concentration, junction geometry, and flow rates (FR) were investigated to determine their effects on the size of generated particles both experimentally and using predictive ANN models. The designed ANN models in this study illustrates the possibility of rapid and efficient generation of PLGA microparticles in silico with tunable sizes even when various microfluidic systems are combined.

## Results

This work presents a new machine learning application for prediction of PLGA microparticle sizes generated by microfluidic devices. ML microparticle size prediction models dedicated to three individual devices as well as more general models predicting multiple device characteristics are demonstrated. Microfluidic devices ensure droplet monodispersity and subsequent uniformity of microparticles sizes. The final ANN structures as well as the optimum activation functions for each ANN model are shown in Table1.

**Table 1:** The final ANN structures, optimum activation functions, correlation coefficients, and errors obtained from the developed ANN models.

ANN model	ANN structure (input-hidden-output layers)	Hidden activation function	Output activation function	Correlation coefficient (r^2^)			Error		
				Training dataset	Test dataset	Validation dataset	Training error	Test error	Validation error
ANN-A	5-7-1	Tanh	Identity	0.988	0.988	0.986	3.29	2.57	3.80
ANN-B	5-4-1	Exponential	Identity	0.998	0.990	0.994	0.54	5.23	4.94
ANN-C	5-10-1	Exponential	Tanh	0.999	1	1	0.48	0.88	0.83
ANN-AB	7-12-1	Exponential	Exponential	0.969	0.945	0.948	9.05	13.49	17.09
ANN-ABC	8-9-1	Exponential	Exponential	0.978	0.975	0.971	6.23	6.45	9.46

### ML model A—single emulsions: formation of PLGA microparticles using a single-junction chip

**Figure 2:**
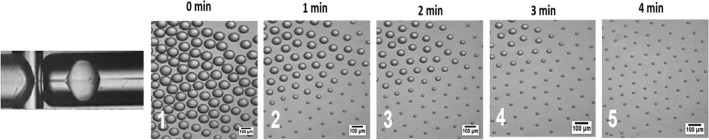
Morphology of the PLGA droplets and microparticles obtained via the microfluidics technique in junction (in-chip, first image left), from left to right (off-chip): (1) before, (2–4) during, (5) and after DMC evaporation. Images show the narrow distribution of PLGA microparticles sizes after polymerization. Further, the size of PLGA droplets was 95.23 ± 1.79 µm and reduced to 31.29 ± 1.08 µm after DMC evaporation as calculated using Image J software, version 1.52a (NIH, US) [https://imagej.nih.gov/ij/].

PLGA microparticles were produced by combining a microfluidic 3D flow focusing droplet generation device containing a single droplet producing nozzle with solvent evaporation. The size of PLGA containing droplets were determined in-chip and the size of PLGA particles were subsequently determined following DCM evaporation. Generation of droplets and resulting particles with different sizes were achieved by varying the aqueous and DCM flow rates were inspected by microscopy (Fig. 2).

**Figure 3:**
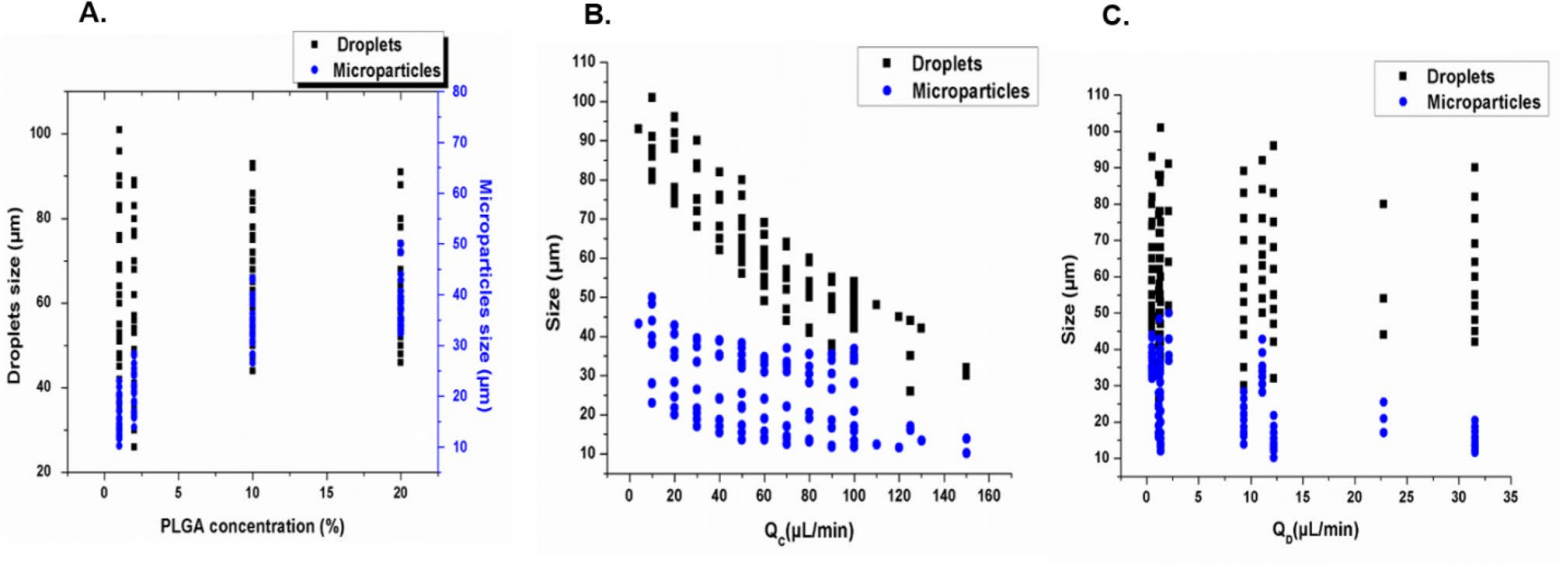
Mean PLGA droplet and microparticle sizes resulting from MFS A for various conditions. Droplets generated by the microfluidic 3D flow focusing droplet device with a single junction. Mean droplet and particle sizes versus four different concentrations of PLGA (1, 2, 10, 20%) (**A**); different flow rates of the continuous phase (Q_C_) (**B**); and different flow rates of the disperse phase (Q_D_) for PLGA solution (**C**).

Generation of droplets of a controlled size for particle production can be easily achieved by the microfluidic device while adjusting the PLGA concentration and controlling the flow rates of the continuous and disperse phases. Increasing PLGA concentrations (from 1 to 20%) led to larger droplets and subsequent particles (Fig. 3A). At 1% PLGA, in the disperse phase the sizes of droplets generated ranged from 35 to 101 µm depending on input flow rates. The corresponding particles after DMC evaporation was 12–23 µm. Furthermore, droplets sizes were 26–88, 44–86, 46–48 µm for 2, 10, 20% of PLGA concentrations and produced microparticles with sizes of 16–28, 28–40, and 35–44 µm, respectively. To investigate the influence of flow rate on droplet and particle size we determined the resulting sizes in response to various flow rates for the continuous as well as the disperse phase with the four different concentrations of PLGA (1, 2, 10, 20%). Droplets and resulting particle sizes decreased with increasing aqueous phase flow rate and decreasing PLGA disperse phase flow rate. Contrarily, larger droplets and particles were generated at lower aqueous phase and higher PLGA dispersed phase flow rates (Fig. 3B,C). The diameters of the droplets and particles were tuned by changing the flow rates of the two phases, between 10–150 µL/min for the continuous phase and 0.5–31.5 µL/min for the disperse phase. Outside of these ranges, the system did not produce droplets, but instead resulted in single phase or co-laminar flow.

**Figure 4:**
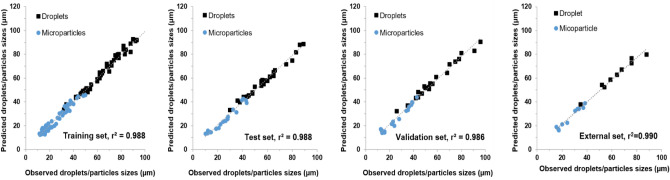
Correlation between observed and predicted droplet/particle size generated by 3D flow focusing droplet chip with a single junction. Results obtained from the developed ANN-A model implemented by Statistica v13.3. Results shown for the developed ANN-A model for the training, test, validation, and external datasets. The reported r^2^ values were calculated for each dataset including droplets and microparticles data.

Training of the ANN model (i.e., ANN-A) were performed using varied PLGA concentrations, flow rates for both aqueous and disperse phase as input variables. The resulting sizes of both droplets and particles as a function of these input variables that were generated by this single- junction chip were used as the outputs of the ANN model. The optimum ANN structure used, the correlation coefficients between predicted and measured droplet and particle sizes, and the error for all datasets used in training, testing and validating ANN-A are shown in Table 1. It can be noted that the ANN model developed here is simple in structure and provided highly accurate results. The overall correlation coefficient (r^2^) between the actual (experimentally observed) and the predicted size values of model ANN-A was 0.988 with residuals randomly scattered and typically lying in the range of ± 5 μm (Fig. S1). The overall r^2^ value represents the correlation coefficient between observed and predicated values for the training, test, and validation datasets for all data including droplets and microparticles. Also, the correlations between observed and predicted values for the individual datasets used were r^2^ = 0.988, 0.988, and 0.986, for training, test, and validation datasets, respectively (Fig. 4). Validation of the developed ANN was confirmed by the high correlation between observed and predicted values of the data in the test and validation datasets. The trained and validated ANN models developed here were subsequently employed in a further step to test an external dataset where the predictions of the sizes of the droplets or particles are required. This was performed using experimental data for 20 new experimental conditions, not used for the prior training, testing or validation of the model. The results showed highly accurate predictions with r^2^ = 0.99.

Interrogation of the network sensitivity analysis offered quantitative assessment of the relative importance of the various input features in determining the sizes of the droplets and particles. By this means, it was found that the order of importance of the input features for the ANN-A model was: aqueous phase flow rate > PLGA concentration > PLGA flow rate > droplet/particle classifier.

### ML model B—prediction of PLGA microparticle sizes in a microfluidic 7-junctions device

**Figure 5:**
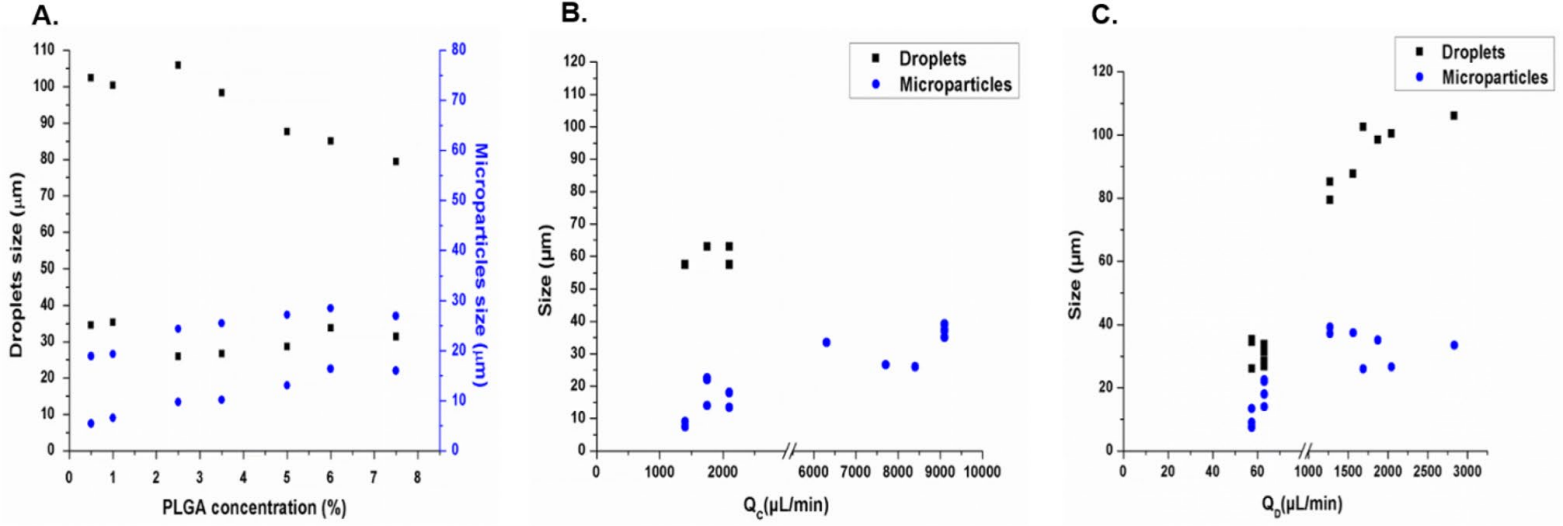
The experimental results of the size distribution of the PLGA droplets/particles before and after polymerization and solvent evaporation in various conditions. Droplets generated by the 3D flow focusing droplet chip with 7 junctions. Droplets and particles size versus different concentrations of PLGA (0.5, 1, 2.5, 3.5, 5, 6, 7%) (**A**), the flow rates of the continuous phase (**B**), and the dispersed phase (**C**).

Figure 5 shows the variation of the droplets and microparticle sizes as the PLGA concentration increases. The used chip resulted in generation of PLGA droplets with sizes ranging from 26 to 102.5 µm while microparticles have sizes of 7–40 µm.

**Figure 6:**
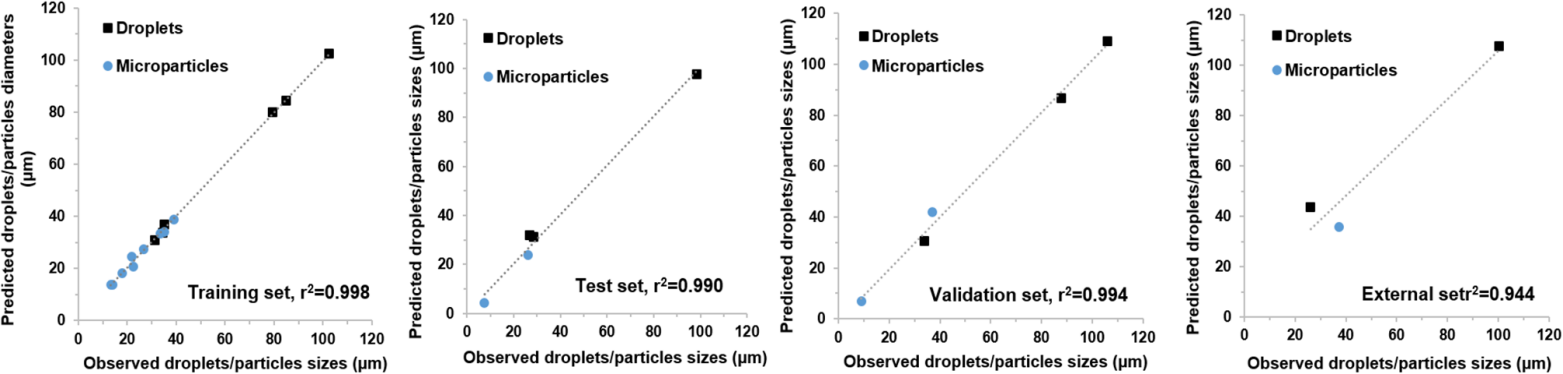
Correlation between experimentally observed and predicted droplet/particle size generated by the flow focusing droplet chip with 7 junctions operating in parallel. Results obtained from ANN-B model implemented by Statistica v13.3. Data shown for Training, test, validation, and external datasets. The reported r^2^ values were calculated for each dataset including droplets and microparticles data.

Similar to MFS A, MFS B was used to train an ANN model using the experimental data, and termed ANN-B (Fig. 6). The optimum ANN structure used in addition to the correlation coefficients of all datasets are shown in Table 1. It can be noted that the ANN model developed here is simple in structure and provided highly accurate results. The correlation coefficient (r^2^) between the actual (experimentally observed) and the predicted size values of ANN-B model was 0.995 with residuals randomly scattered and typically lying in the range of ± 5 μm (Fig. S2). Also, the correlations between observed and predicted values for the individual datasets used were r^2^ = 0.998, 0.990, and 0.994, for training, test, and validation datasets, respectively. Validation of the developed ANN was confirmed by the high correlation between observed and predicted values of the data in the test and validation datasets. The trained and validated ANN model was subsequently employed in a further step to test an external dataset where the predictions of the sizes of the droplets/particles are required using this chip. This was performed using experimental data for three new potential experimental conditions. The results showed highly accurate predictions as shown in Fig. 5. Additionally, interrogation of the network sensitivity analysis showed that the order of importance of the input features for the ANN-B model was: aqueous phase flow rate > PLGA flow rate > PLGA concentration > droplets/particle classifier.

### ML model C—prediction of PLGA microparticle formation from multiple emulsions in two single-junction chips

**Figure 7:**
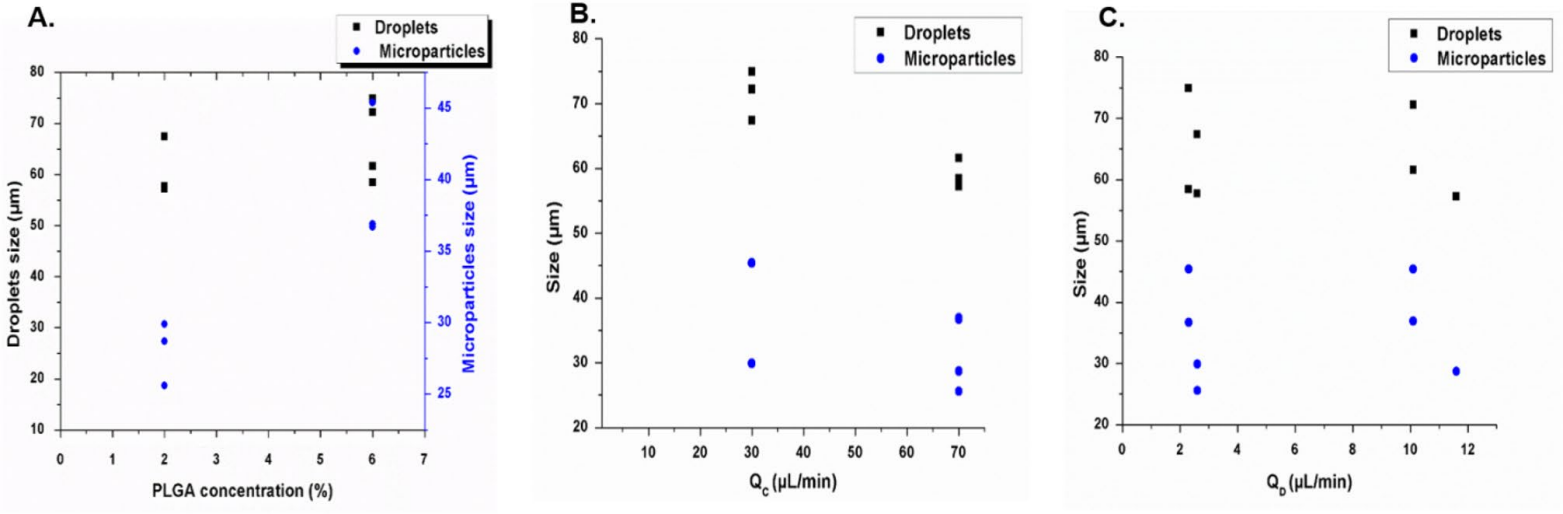
The experimental results of the size distribution of the final PLGA multiple emulsions (W/O/W) before and after polymerization and solvent evaporation in various conditions. Droplets generated by two flow focusing chips placed in series. Droplets and particles size versus two concentrations of PLGA (2, 6%) (**A**), the flow rates of the continuous aqueous phase (**B**), and the dispersed phase(PLGA/DCM/surfactant) (**C**).

**Figure 8:**
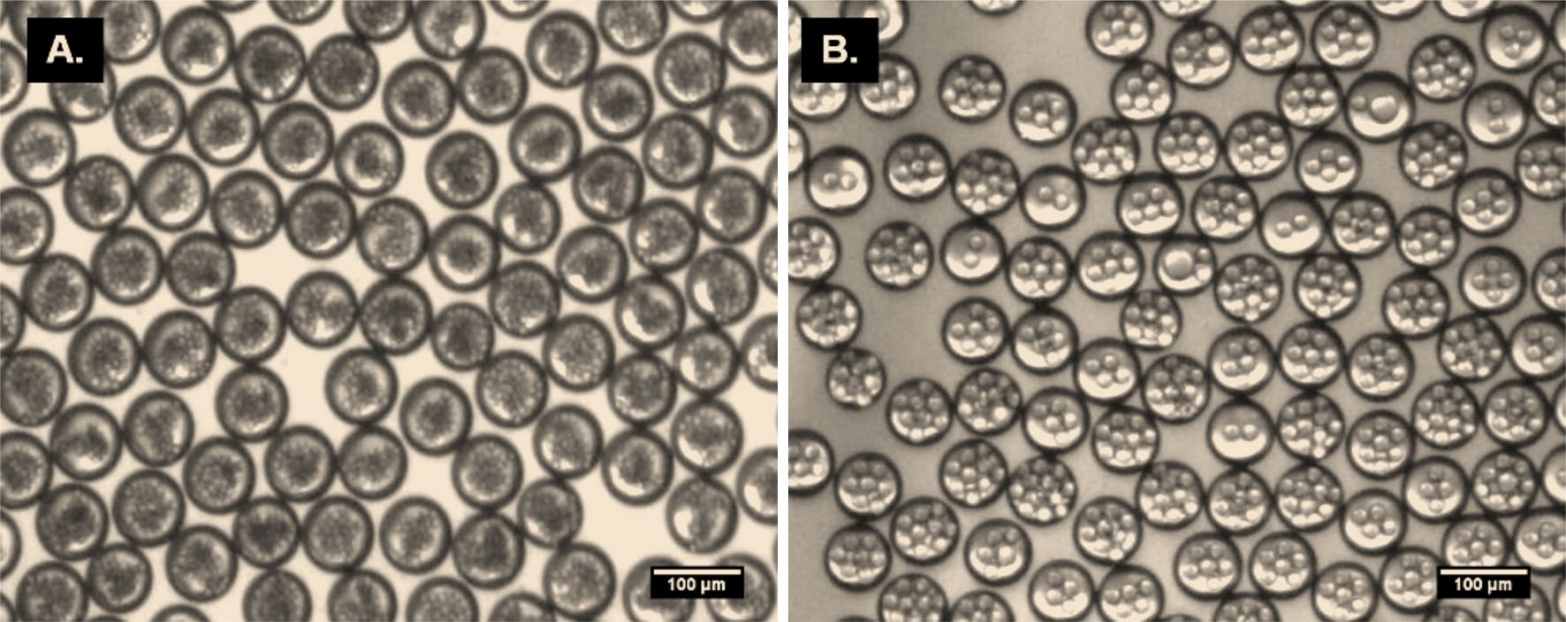
Morphology of the 50 µm PLGA W/O/W droplets before DMC evaporation with 3 µm (**A**) and 14 µm (**B**) inner water droplets.

Fabrication of multiple emulsions of PLGA microparticles was performed by two consecutive cross-junctions featuring flow focusing microfluidic chips placed in series. The size of the generated inner and outer droplets were controlled by adjusting the PLGA concentrations and the flow rates of continuous and dispersed phases. The sizes of the inner water droplets are between 3 and 14 µm while the outer core–shell PLGA droplets are between 57 and 72 µm. The final size of W/O/W emulsions after DMC evaporation are ranges from 30 to 45 µm. Both the resulting size of droplets and particles are dependent on PLGA concentrations and flow rates (Fig. 7). Further, the number and size of inner water droplets in PLGA shell can be controlled by changing the flow rate conditions. In the first chip, a high PLGA flow rate results in a greater number of water droplets in each PLGA droplet. In the second chip, higher aqueous phase flow rate results in an increase in the final droplets size (Fig. 8).

**Figure 9:**
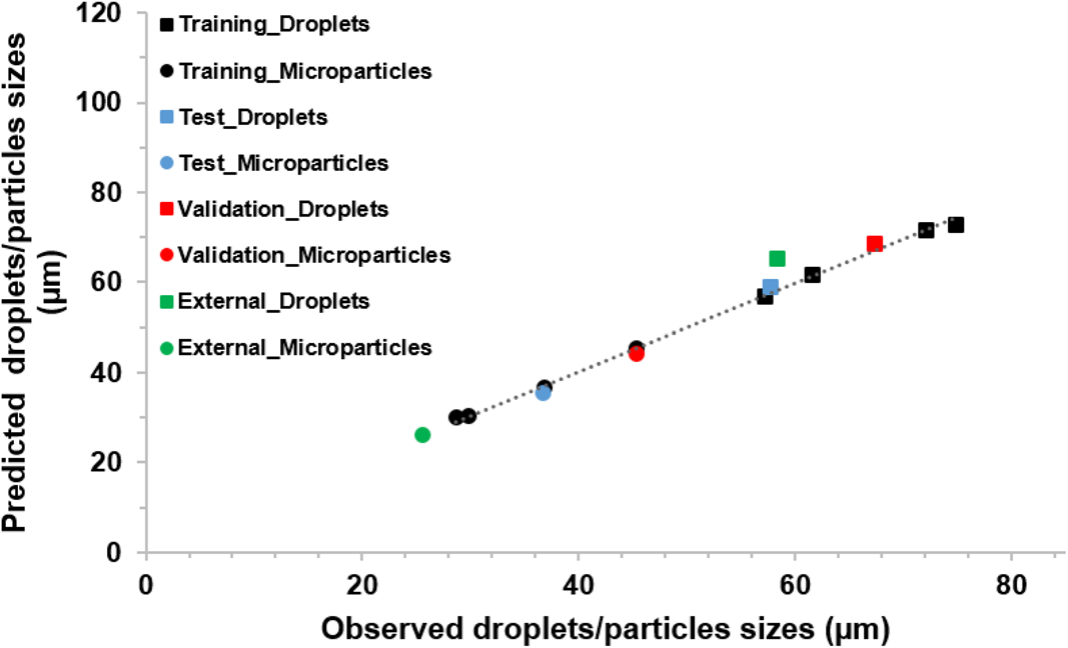
Correlation between observed and predicted droplet/particle size generated by two consecutive cross-junctions featuring flow focusing microfluidic chips placed in series obtained from ANN-C model implemented by Statistica v13.3. Data are shown for training, test, validation, and external datasets.

Similar to the previously trained ANN models above, an ANN model was trained using these data, accordingly and labeled as ANN-C. The optimum ANN structure used in addition to the correlation coefficients of all datasets are shown in Table 1. It can be noted that the overall correlation coefficient as well as the individual correlation coefficient values for training, test, and validations datasets are very high which may be attributed to the small size of data (total 12) that may lead to overfitting. However, validation of the developed ANN was confirmed by the high correlation between observed and predicted values of the data in the external datasets (Fig. 9). Additionally, residuals randomly scattered and typically lying in the range of ± 2 μm (Fig. S3). Examination of the network sensitivity analysis showed that the order of importance of the input features for the ANN-C model was: PLGA concentration > aqueous phase flow rate > PLGA flow rate > type (droplets/particles).

### ML model AB—a single in silico model for prediction of PLGA microparticles generated by ML models A and B

To assess the ability of ANNs to predict PLGA droplet/particle sizes when different microfluidics approaches jointly combined, one ANN model consisting of data obtained from models A and B for single emulsions production was developed. Good correlation between observed and predicted values was obtained for training, test, and validation data sets with r^2^ values of 0.969, 0.945, and 0.948, respectively. In addition, r^2^ value for the external dataset was 0.972. Five variables were used for training; the relative importance of these variables to determine the aimed output was in a decreasing order: chip type > aqueous phase flow rate > PLGA flow rate, PLGA concentration, and droplets/particles.

### ML model ABC—a single in silico model for prediction of PLGA microparticles generated by ML models A, B, and C

**Figure 10:**
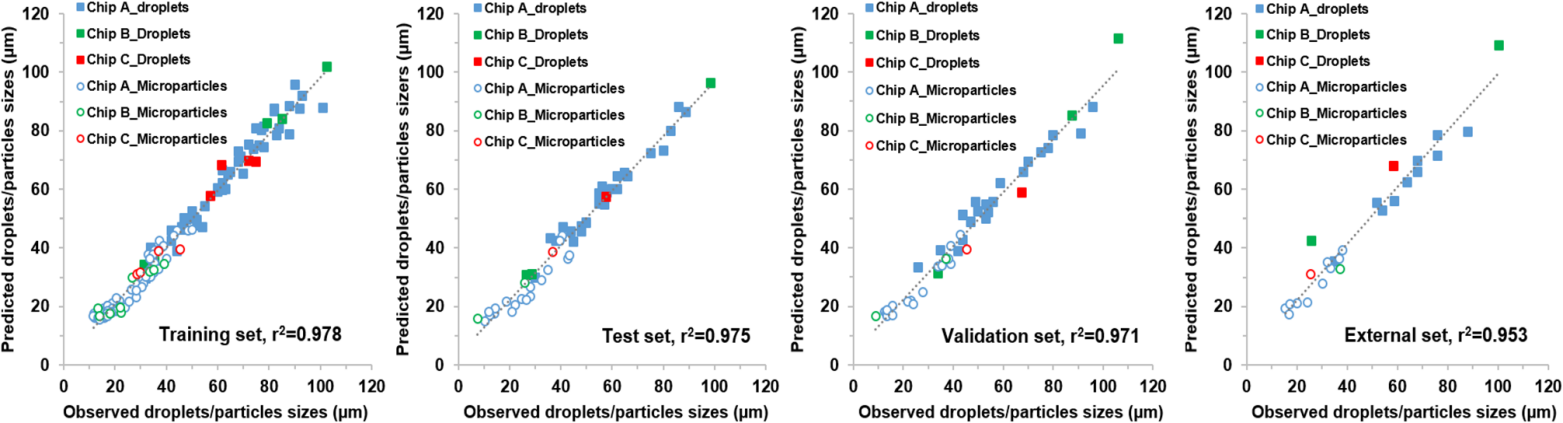
Correlation between observed and predicted droplet/particle sizes with the corresponding r^2^ values obtained from the developed ANN-ABC model implemented by Statistica v13.3. Correlations are shown for the training, test, validation and, external datasets for three types of microfluidic systems: MFS A, MFS B, and MFS C. The reported r^2^ values were calculated for each the dataset including droplets and microparticles data.

An ANN model consisting of the data from A, B, and C models was developed (Fig. 10). The optimum final ANN structure consisted of 8, 9, 1 for input, hidden, and output neurons, respectively. The trained model provided highly accurate predictions with r^2^ values for training, test, and validation datasets equal 0.978, 0.975, and 0.971, respectively. In addition, r^2^ value for the external dataset was 0.953. Finally, the relative importance of the input variables on the prediction of droplet/microparticle sizes was, in decreasing order: chip type > PLGA flow rate, aqueous phase flow rate > PLGA concertation > type (droplets/particles).

**Figure 11:**
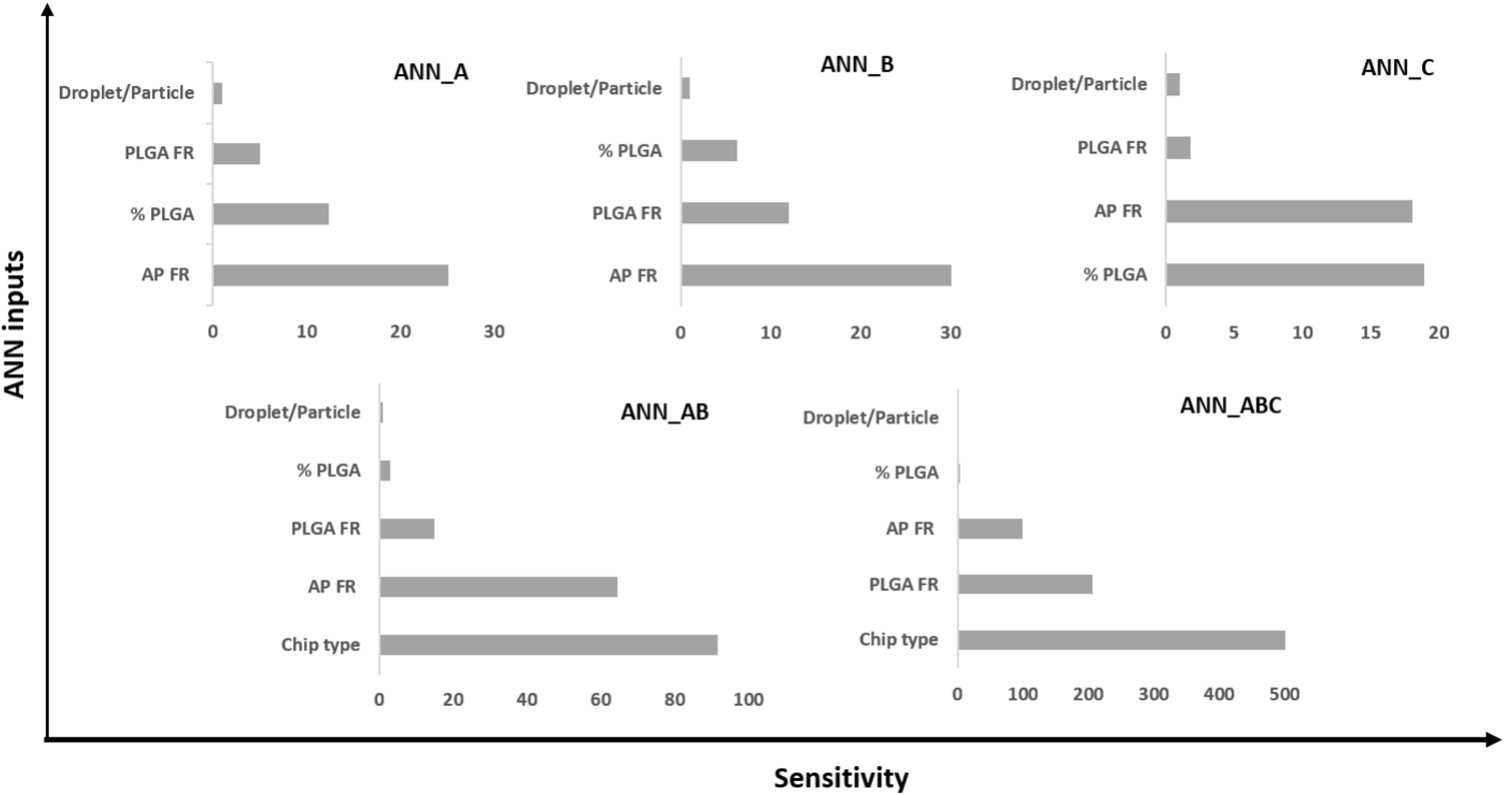
Sensitivity analysis of the five ANNs developed in this study. Each bar represents the relative importance of the specific input feature to the neural network output. Note that ANN sensitivity analysis for the chip type in ANN-ABC lies beyond the X-axis scale (> 5000) and is not represented here for clarity.

In addition, interrogation of the selectivity analysis results provided insightful information on the influence of these variables on PLGA droplet/microparticle sizes (Fig. 11). Although the flow rate of the aqueous phase was an important determinant factor for the output for all single-chip ANN models, chip type was the most important determinant in models ANN-AB and ANNN-ABC. However, the ANN-ABC model which consists of more diverse data provided a new insight on the substantial importance of the flow rate of the dispersed phase on determining the PLGA droplet and microparticle sizes.

## Discussion

We report on a new application of AI technology for predicting the sizes of monodisperse PLGA microparticles generated by microfluidics. The training of ANN models was based on generation of PLGA microparticles either as single or multiple emulsions by flow focusing microfluidics. The relationships between PLGA droplet/particle size and PLGA concentrations, continuous and dispersed phases, and flow rates were investigated while taking into account that the microfluidic chips used exhibited different characteristics involve hydrophilicity, number of junctions, and chip geometry. Subsequently, the experimental data were used to develop different ANN models that can predict the sizes of the resulting droplets and particles in silico based on key factors controlling the generation of microparticles in microfluidic devices.

Three different microfluidic approaches were developed in the current study to generate PLGA microparticles. The first approach aimed to create single PLGA microparticles utilizing a single junction 3D flow focusing chip with hydrophilic channels. Similarly, the second approach aimed to create single emulsions of PLGA microparticles utilizing a 3D flow focusing chip with seven parallel hydrophilic channels to achieve the highest possible throughput. The third approach aimed to create core–shell PLGA particles utilizing two single flow focusing junction chips with different hydrophilicities, in series. First water emulsion droplets were generated in PLGA/DCM solution in the first hydrophilic chip and then delivered via FEP tubing to the second hydrophobic chip to generate core–shell PLGA droplets. In all these experimental models, generation of droplets and subsequently particles of a controlled size were achieved in the microfluidic devices while adjusting the PLGA concentrations and controlling the flow rates of continuous and dispersed phases at the chip junction(s). The sizes and characteristics of the droplets and microparticles produced in these microfluidic devices depend on a number of parameters such as PLGA concentration, two or three input flow rates and the characteristics of the microfluidic devices. The relationships between these input parameters and the resulting droplet and particle size is complex with distinguishably non-linear features. The capacity of ANNs to generalize and analyze complex nonlinear relationships make ANNs suitable to model the complex and non-linear relationships in microfluidic droplet and microparticle production^9,31^. This study demonstrates a good generalization and pattern recognition ability in the developed ANN models.

We systematically developed a number of ANN models for in silico prediction of PLGA microparticle sizes formed by different microfluidics systems by training several ANNs on experimental data generated using three above-mentioned microfluidic chip types. Initially, each chip-related dataset was used to build a separate ANN model for each individual device. This approach resulted in the development of three ANN models (ANN-A, ANN-B, AND ANN-C) which can *individually* predict—with high accuracy—PLGA droplet/microparticle sizes generated by each microfluidic device. Subsequently, experimental data for two *single* microfluidic systems were used to train a more general ANN model (ANN-AB). The result was a *single* ANN model that is capable of predicting with high accuracy the sizes of droplets and microparticles formed by any of these microfluidic devices.

After successful development of these neural network models, a final step was carried out to build a general in silico model capable of predicting PLGA droplet and microparticle sizes generated by three microfluidics systems using a *single* ANN model. This model (named as ANN-ABC) is simple in structure with minimal required number of variables (i.e., chip type, PLGA concentration, flow rates of aqueous phase and dispersed phase, and types (droplet or particle)).

This work is to our knowledge the first to report an AI/microfluidics application utilizing several microfluidics systems to develop a multi-purpose predictive model in a single in silico platform. In addition to providing accurate predictions for three different microfluidic devices, the trained ANN models demonstrate the potential extendibility of this approach, both with regard to different polymers, droplet contents and droplet producing microfluidics device design.

## Conclusion

This study presents a new promising application of AI for rapid fabrication of PLGA microparticles with numerous sizes using different microfluidic systems. This approach can save substantial time and effort in tunable particles generation. Five different in silico models were developed based on data extracted from three different microfluidic systems. The systematic development of ANN models ultimately resulted in a single in silico model which could accurately predict the sizes of polymeric microparticles produced by three different microfluidic systems. The developed ANN models provided highly accurate predictions as well as significant insights into the key determinants required for PLGA microparticle fabrication using microfluidics including flow rates, polymer concentration, and chip design. In addition to PLGA particles, this AI application can be extendable by exploring other types polymeric particles, and can eventually be used for well-controlled production of polymeric particles. Further investigations on integration of AI technologies with microfluidics may offer numerous opportunities for developing efficient and economical production as well as exploring future AI applications using microfluidics in biomimicry and pharmaceutical industry applications.

## Methodology

### Chemical and materials

All microfluidic chips used were purchased from Dolomite Microfluidics (Royston, UK). PLGA (lactide:glycolide 75:25, Mw 76,000–115,000) (719927) and Dichloromethane (DCM) (34856) were purchased from Sigma Aldrich, PW11 surfactant blend (7206001) from ParticleWorks, and Aqua-Phase contains 1–5% proprietary polymer blend (3200774) from Dolomite. Fluids were injected into the microfluidic devices by Mitos P-Pumps (Dolomite, 3200016) and in-line Mitos Flow Rate Sensors (Dolomite, 3200097 and 3200098) were used to monitor the flow rates. The aqueous flow was split off the chip using T-connector (Dolomite, 3000397). The size of droplets was tracked in real time under a high speed microscope (Dolomite, 3200531) focused at the chip junction of the microfluidic chip.

### Experimental systems

Three microfluidic systems were assembled and optimized in the lab to generate PLGA microparticles or PLGA core–shell microparticles. In all microfluidic systems, glass microfluidics flow focusing devices were used. Droplet formation was monitored at the microfluidic channel junction using a software controlled high-speed imaging system. The obtained images were used to estimate the size and uniformity of the generated droplets.

#### Microfluidic system A (MFS A)

A 3D flow focusing microfluidic device with 100 μm channels and surface treated for hydrophilicity (Dolomite, 3200433) was used to create PLGA droplets. The depth of the channels at the flow-focusing junction is 100 μm and the width is 105 μm. The chip has three inlets and one outlet (Fig. 1A). An aqueous solution serving as the continuous phase were injected into two inlets, while a solution of PLGA dissolved in DCM serving as the disperse phase was injected into the central channel of the device. The stream of PLGA solution broke up at the channel junction to generate monodisperse PLGA droplets due to generation of shear force at the narrow orifice. The droplets were collected at the outlet in the aqueous solution. The aqueous suspension contained 2% PW11 surfactant to lower surface tension and limit coalescence of the droplets. The collection vessel was pre-loaded with aqueous solution to minimize aggregation and to dilute the particles. To create PLGA microparticles, droplets were solidified by evaporating DCM solvent which enabled conversion of droplets to particles within a few minutes and leads to a decrease in the size of droplets. Parametric characterization involved the effects of PLGA concentrations, and the flow rates of continuous and dispersed fluids on the size of PLGA droplets/particles were practically investigated.

#### Microfluidic system B (MFS B)

A 3D flow focusing microfluidic device similar to MFS A, except that the microfluidic chip used has seven parallel junctions (Telos 3D flow focusing device, Dolomite, 3200819). The channels are 100 μm wide and surface treated for hydrophilicity. The depth of the channel at the flow-focusing junction is 54 μm and the width is 75 μm. The Telos manifold equally distributes the two fluids, PLGA dissolved in DCM and the aqueous phase to the respective inlet channels across the chip to create monodisperse droplets of PLGA polymers in the continuous aqueous phase (Fig. 1B). A range of PLGA concentrations from 0.5 to 7.5% were injected to generate PLGA microparticles with a range of sizes. Continuous and dispersed phases were injected at substantially higher flow rates in the MFS B parallel junctions device than single microfluidic junction device. The flow rate range used in MFS B were approximately a factor of 70 higher than those used in MFS A.

#### Microfluidic system C (MFS C)

This system aimed to generate multiple emulsions using two microfluidic devices arranged in sequence (Fig. 1C). First a 14 μm microfluidic device with a flow focusing junction geometry with 14 × 17 μm (depth × width (Dolomite, 3200137) channels surface treated for hydrophobicity were used, and the resulting aqueous droplets in DCM/PLGA were subsequently routed to the device used in MFS A. Core–shell microparticles with aqueous cores and PLGA shell were generate in two steps. The first step involved the emulsification of water as the dispersed phase in the continuous phase composed of PLGA/DCM with 0.5wt% CRODA Synperonic PE/F68 added as a stabilizer, followed by the emulsification of this primary emulsion in a second aqueous phase. The inner droplets W/O single emulsions were passed through tubing (0.25 mm ID FEP tubing) connecting the two microfluidic devices. The final W/O/W emulsions were composed of an inner phase made of water, a middle phase of PLGA in DCM with 0.5 wt% CRODA Synperonic PE/F68 and an outer phase made of Aqua-Phase.

In all experimental microfluidic systems, PLGA droplets were collected in the continuous phase at the outlet in order to allow indirect and slow evaporation of DMC which firstly dissolved into the aqueous phase, and then evaporated from the water–air interface resulting in a conversion of droplets to particles. In the case of multiple particles, DMC rapidly evaporated at the thin droplet edge and slowly within the droplet center where the continuous phase forms a thicker layer. Finally, solidified particles were washed with water and imaged.

### In silico models

Multilayer perceptron (MLP) ANNs were implemented using Statistica, version 13.3 software^32^. Five ANN models were developed and labelled as ANN-A, ANN-B, ANN-C, ANN-AB, and ANN-ABC. The first two models (ANN-A and ANN-B) were trained to predict the droplet and resulting microparticle sizes of two *single* PLGA microparticle producing microfluidic systems (MFS A and B), *individually*. ANN-C was trained to predict the droplet and resulting microparticle sizes of a multiple emulsions producing microfluidic system (MFS C), *individually*. ANN-AB is an ANN model developed to predict the droplet and resulting microparticle sizes of the two *single* PLGA microparticle producing microfluidic systems (MFS A and B), *simultaneously*. Finally, ANN-ABC was trained to predict the droplet and resulting particle sizes of all three microparticle producing microfluidic systems (MFS A, B, and C), *simultaneously*. A total of one thousand MLP-ANNs were trained for each model. Only ANNs with the highest correlation coefficients (r^2^) and lowest error (obtained using the sum of squares (SOS) error function) for each target device or set of devices were retained. The obtained errors were used as an indication of the accuracies of the predictive models.

Input data consisted of the experimental data corresponding to each microfluidic system together with different features related to the size and the type (i.e., droplets or particles) of the generated monodisperse single, and multiple PLGA droplets. These data consisted of PLGA concentration, flow rates of continuous and dispersed phases, types (i.e., droplet or particle). For the combined models (i.e., ANN-AB and ANN-ABC), an additional classification feature was included, namely microfluidic device type. Only relevant features were used to design the ANN models in order to keep ANN models simple, improve their generalization and pattern recognition capability, as well as to reduce the training time. All continuous variables were normalized to the 0–1 range.

The obtained data from the experimental microfluidic approaches was subsequently used to train, test and validate the machine learning models. The total numbers of data points in each dataset used for each model was 186, 25, 12, 211, and 223 for models ANN-A, ANN-B, ANN-C, ANN-AB, and ANN-ABC, respectively. The data in each model has been divided into three datasets: training (~ 60%), testing (~ 20%), and validation (~ 20%) datasets. Furthermore, a number of additional datasets, 20, 3, 2, 23, and 25 for models ANN-A, ANN-B, ANN-C, ANN-AB, and ANN-ABC, respectively were used as external datasets. The external dataset include data that have not been seen by the ANN model previously, neither in training, nor test or validation. The distribution of the inputs into these datasets was carried out in a way to consider the limits and the diversities of the data. Learning parameters were optimized to improve the prediction capabilities of the models. This included investigating four activation functions for both hidden and output neurons including identity, logistic, tanh, and exponential activation functions. The number of nodes in the hidden layers were optimized using a trial-and-error approach. The output layer consisted of the results of the experimentally measured droplet sizes.

## Supplementary information

Supplementary Information.
