# Supplementary material for: Artificial intelligence application for rapid fabrication of size-tunable PLGA microparticles in microfluidics
Source: Sci Rep. 2020 Nov 11;10:19517. doi: 10.1038/s41598-020-76477-5 (PMC7658240; doi:10.1038/s41598-020-76477-5)
Supplement: Supplementary file 1 — Supplementary Information. [file 41598_2020_76477_MOESM1_ESM.pdf]

**Supplementary material**

# Artificial Intelligence Application for Rapid Fabrication of Size-tunable PLGA Microparticles in Microfluidics

Safa A. Damiati [1,*], Damiano Rossi [2,3], Haakan N. Joensson [4,5], Samar Damiati [6,7,*]

[1] Department of Pharmaceutics, Faculty of Pharmacy, King Abdulaziz University (KAU), Jeddah, Saudi Arabia
[2] University College London, Department of Chemical Engineering, Torrington Place, London WC1E 7JE, UK
[3] Blacktrace Holdings Ltd (Dolomite Microfluidics), Royston SG8 5TW, UK
[4] Division of Protein Science, KTH Royal Institute of Technology, Stockholm, Sweden
[5] Novo Nordisk Foundation Center for Biosustainability at KTH, Stockholm, Sweden
[6] Department of Biochemistry, Faculty of Science, King Abdulaziz University (KAU), Jeddah, Saudi Arabia
[7] Division of Nanobiotechnology, Department of Protein Science, Science for Life Laboratory, School of Engineering Sciences in Chemistry, Biotechnology and Health, KTH Royal Institute of Technology, Stockholm, Sweden

* Corresponding authors: S.A.D.: smdamiati@kau.edu.sa; S.D.: samar.damiati@scilifelab.se

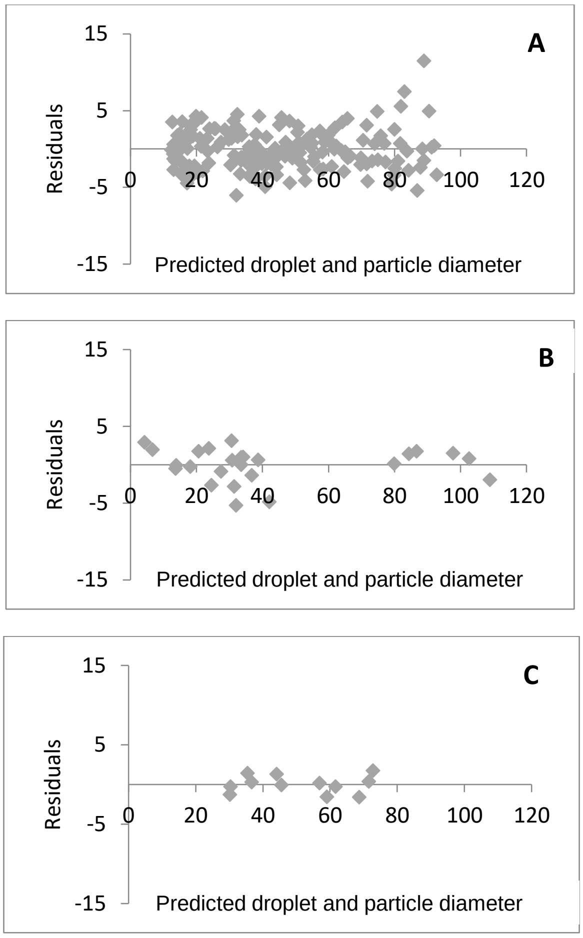


**Figure S1.** Plots of residuals for regression of predicted *vs*. observed droplets and particles sizes using three ANN models: ANN-A model for MFS A, ANN-B for MFS B, and ANN-C for MFS C.

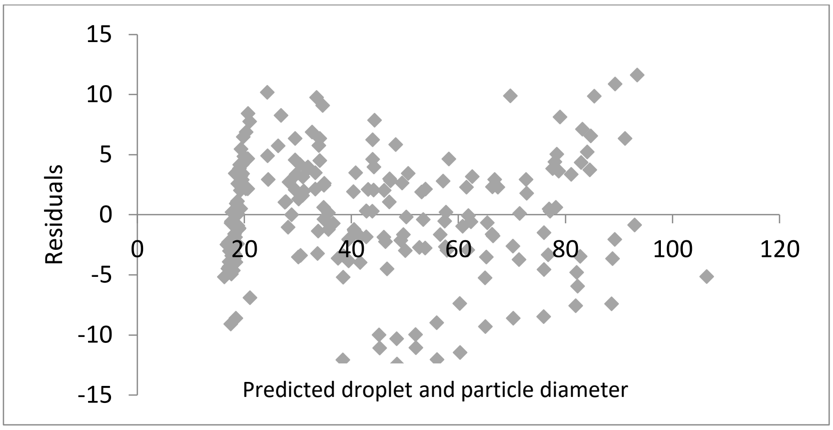


**Figure S2**. Plot of residuals for regression of predicted *vs*. observed droplets and particles sizes using one ANN trained using combined data of MFS A and MFS B.

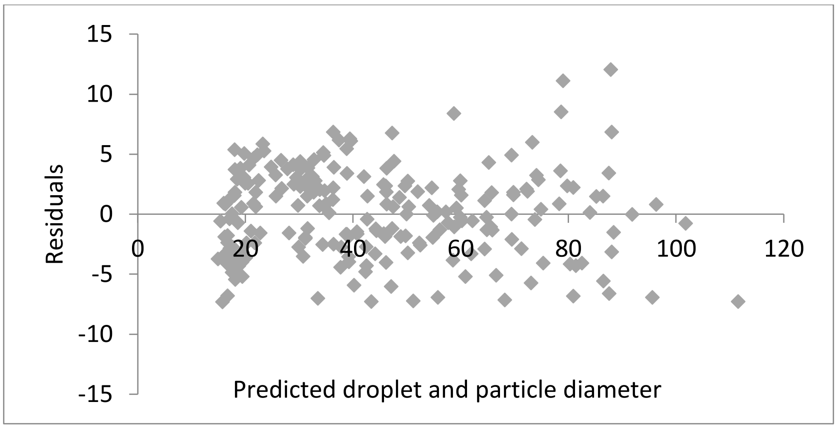


**Figure S3**. Plot of residuals for regression of predicted *vs*. observed droplets and particles diameters using one general ANN trained using combined data of MFS A, MFS B, and MFS C.
